# Multi-Stage Robust Bayesian High-Resolution Identification of Asynchronous Blade Vibrations Using Blade Tip Timing

**DOI:** 10.3390/e28050505

**Published:** 2026-04-30

**Authors:** Qinglei Zhang, Xiwen Chen

**Affiliations:** China Institute of FTZ Supply Chain, Shanghai Maritime University, Shanghai 201306, China; qlzhang@shmtu.edu.cn

**Keywords:** Blade Tip Timing, asynchronous vibration, multi-stage identification, robust Bayesian estimation, high-resolution frequency estimation, dynamic window

## Abstract

Blade Tip Timing (BTT) is an essential non-contact technique for monitoring vibrations in rotating machinery, but its practical accuracy is often degraded by noise, undersampling, and spectral leakage. This paper proposes a multi-stage robust Bayesian high-resolution identification framework that systematically addresses these challenges. A recursive digital algorithm based on Kalman filtering estimates the rotational speed without requiring once-per-revolution probes, effectively suppressing sensor noise. An attention-enhanced dynamic convolutional autoencoder then generates channel-specific window functions to minimize spectral leakage. The core identification algorithm extracts phases via all-phase FFT and employs sub-bin interpolation to overcome the resolution limitation of conventional FFT. A Tukey-biweight-based robust aggregation strategy is used to suppress the influence of abnormal or unequal-quality sensor channels during multi-channel phase fusion. A Bayesian prior distribution over the vibration order guides the estimation toward physically plausible values under noisy conditions. Finally, a coarse-to-fine multi-stage search strategy drastically reduces computational burden while preserving accuracy. Experiments on a rotor-blade test bench at constant and variable speeds show that the method reduces the noise floor by about 60 dB, achieves a maximum frequency identification error of 7.84%, and accelerates the search by approximately 48.6% compared to exhaustive search. The proposed method provides a reliable and efficient solution for blade health monitoring.

## 1. Introduction

Blades are critical components in turbomachinery such as compressors and gas turbines. They operate under severe vibration, centrifugal, and thermal loads, making condition-based maintenance supported by reliable online health monitoring essential to extend their useful life and ensure safe operation [1]. Vibration monitoring techniques can be divided into contact and non-contact categories. Traditional contact measurement involves complex preparation, low signal transmission reliability, and may alter the mechanical characteristics of the blade [2]. Non-contact techniques, in contrast, offer ease of use, low installation cost, high sensitivity, and the ability to monitor the whole blade circle simultaneously [3]. Recent reviews of the literature indicate that BTT research has evolved from basic non-contact vibration measurement toward a more integrated framework involving sensor design, probe-layout optimization, signal processing, uncertainty analysis, and structural health monitoring applications. In particular, Wang et al. provided a systematic review of BTT technology for aeroengines, covering the theoretical foundations of BTT, commonly used sensor types, optimization strategies for sensor placement and sensor number, major signal-processing and parameter-identification methods, as well as the main sources of measurement errors and uncertainties. These studies show that the practical performance of BTT depends not only on the identification algorithm itself, but also on the coupled effects of sensing configuration, undersampling characteristics, signal quality, and uncertainty management [4,5]. Meanwhile, the interpretation of blade vibration signatures also relies on an adequate understanding of blade dynamics and structural modeling. Recent surveys on aero-engine blade dynamics have systematically reviewed lumped-mass, finite-element, and semi-analytical models, together with their associated solution methods and vibration characteristics. In addition, review work on rotating cracked blades has summarized crack propagation mechanisms, open/breathing crack models, and the corresponding dynamic modeling approaches for fault-related vibration analysis. Representative nonlinear dynamic studies, such as the analysis of rotating shrouded blades with impacts, further demonstrate that centrifugal stiffening, spin softening, Coriolis effects, and contact/impact interactions can significantly affect blade vibration responses. These modeling studies provide the physical background for understanding why robust identification methods are required when BTT data are acquired under complex operating conditions [5,6,7].

Blade Tip Timing (BTT) has emerged as an efficient non-contact method for blade vibration monitoring [8]. It employs tip timing sensors to record precise blade passage times, from which instantaneous vibrational displacements are calculated. However, BTT signals are inherently undersampled (the sampling rate equals the rotational frequency) and are contaminated by various noise sources, leading to reduced accuracy compared to contact methods [3]. Moreover, the absence of a high-precision once-per-revolution (OPR) probe further complicates the determination of theoretical arrival times [9].

Against this background, considerable research has focused on two closely related issues in BTT-based blade vibration monitoring: accurate reconstruction of blade arrival-time/displacement information without a dedicated OPR probe, and reliable identification of asynchronous vibration parameters from severely undersampled signals. Russhard proposed a linear fitting approach to generate a fictitious OPR signal [10], while Chen et al. improved the accuracy using a high-precision method (CR-BTT) [11]. The fictitious OPR technique, however, suffers from large errors when rotational speed fluctuates significantly. Alternative methods exploit vibration differences among blades or the same blade at different times [12,13]. Guo et al. used the Levenberg–Marquardt algorithm to identify blade vibrations without an OPR probe [13]. Wang et al. applied a Savitzky–Golay filter to improve BTT estimation under rapid speed fluctuation [14]. Xu et al. developed a recursive BTT algorithm in the angular domain [15]. Wang et al. also proposed a none-OPR BTT-based blade vibration parameter identification method [16]. These approaches aim to accurately calculate the expected blade arrival times, which are essential for deriving vibration displacements.

Once vibration displacements are obtained, parameter identification for asynchronous vibrations is typically performed using spectral methods. The asynchronous vibration frequency ω is related to the rotational frequency Ω by ω=mΩ+Δω, where m is an integer order and Δω is the difference frequency (aliased component). Because ω often exceeds the Nyquist frequency (Ω/2), direct spectral analysis is impossible; instead, the low-frequency Δω is extracted from the undersampled data using the difference-frequency principle [17]. Various techniques have been proposed to determine Δω and m, including autoregressive methods [18], the “5 + 2” approach [19], interpolation methods [20], and least-squares fitting combined with Fourier transforms [21]. Most of these methods rely on Fourier analysis and suffer from the inherent limitations of FFT, such as spectral leakage and the picket-fence effect, especially under non-stationary conditions. Furthermore, they often require a large number of sensors or impose strict constraints on sensor placement.

In recent years, deep learning has shown promise in feature learning and noise suppression [22]. However, pure deep learning approaches may lack interpretability and require large training datasets. For BTT-based blade vibration monitoring, the feasibility of machine learning should be discussed separately for synchronous and asynchronous vibrations. Recent reviews of the literature have pointed out that these two vibration types differ in their under-sampling severity and signal characteristics, which means that different identification strategies are often required. For synchronous vibrations, machine learning can be feasibly used for resonance-region recognition, denoising, feature extraction, or assisting parameter identification when the response patterns are relatively structured. For asynchronous vibrations, however, the problem is generally more difficult because the signals are more severely under-sampled, more sensitive to aliasing and probe-layout effects, and often less supported by abundant labeled data. Therefore, in strongly under-sampled BTT scenarios, pure end-to-end machine learning may be less reliable than hybrid approaches that combine physical signal models with learning-based modules. From this viewpoint, machine learning is more suitable as an auxiliary tool for preprocessing, feature enhancement, or condition classification, while the final parameter identification still benefits from physically interpretable constraints.

Hybrid methods that combine signal processing with learning-based optimization offer a compelling alternative. The present work develops a multi-stage robust Bayesian high-resolution identification framework that systematically integrates several enhancements: (i) a Kalman-filter-based recursive algorithm for accurate speed estimation without an OPR probe; (ii) an attention-enhanced dynamic convolutional autoencoder (ADCAE) that generates adaptive window functions to suppress spectral leakage; (iii) sub-bin interpolation applied to all-phase FFT (APFFT) spectra to overcome the resolution limit; (iv) a Tukey biweight error function for robust aggregation of multi-channel phase information; (v) a Bayesian prior over the vibration order to guide the estimation; and (vi) a coarse-to-fine multi-stage search strategy to reduce computational cost. Experiments on a rotor-blade test bench demonstrate the effectiveness of each component and the overall method.

From this perspective, the contribution of this work is not the mere combination of several advanced techniques, but the construction of a minimally sufficient pipeline for difficult BTT conditions. The recursive digital algorithm improves arrival-time reconstruction when an OPR signal is unavailable; the adaptive window and APFFT/interpolation improve spectral concentration and frequency resolution; the robust cost function mitigates the influence of corrupted channels and channels with unequal data quality; the Bayesian prior constrains the solution to physically plausible orders; and the multi-stage search improves efficiency without changing the estimation target itself. This design principle helps avoid treating the method as a simple accumulation of independent modules.

Although the proposed method contains multiple components, it is not intended as an unnecessarily complicated architecture. Rather, it is a problem-driven framework in which each module is introduced to address a specific limitation of conventional asynchronous BTT identification. In practical OPR-free BTT measurements, several sources of error often occur simultaneously, including rotational-speed uncertainty, spectral leakage caused by undersampling and non-stationarity, sensitivity to abnormal sensor channels, and high computational cost in order search. A single conventional technique typically addresses only one of these issues. Therefore, the proposed framework combines recursive speed estimation, adaptive spectrum optimization, robust phase aggregation, Bayesian regularization, and coarse-to-fine search into a unified pipeline. For clean and stationary cases, simpler signal-processing pipelines may still be sufficient; the full proposed framework is mainly advantageous in noisy, undersampled, OPR-free, and outlier-prone scenarios. The present formulation is intended for constant-speed or quasi-stationary conditions within each analysis window.

## 2. BTT Principles and Asynchronous Vibration Model

### 2.1. Blade Tip Timing Measurement

**Figure 1:**
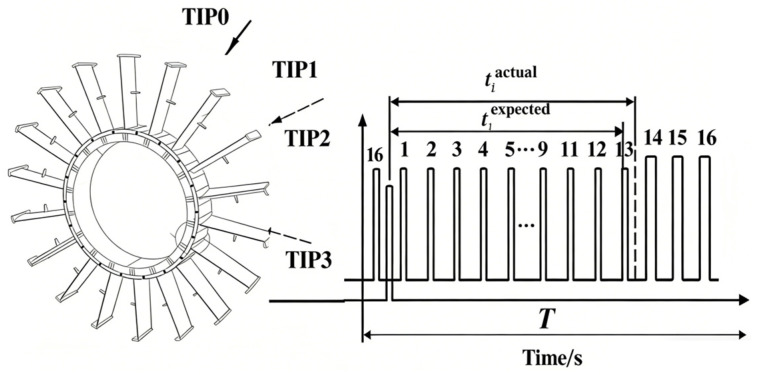
Schematic diagram of the BTT technique.

Figure 1 illustrates the typical configuration of a BTT system. One or more tip timing sensors (e.g., fiber optic, capacitive, or eddy-current) are mounted circumferentially around the blade casing, and a speed synchronization sensor (OPR) may be installed near the shaft to provide a once-per-revolution reference. When a blade passes a sensor, a pulse is generated, and the arrival time $t_{i}^{actual}$ is recorded with high precision. In the absence of vibration, the expected arrival time $t_{i}^{\exp ected}$ is determined solely by the shaft speed, blade tip radius, and sensor angular position. Blade vibrations cause the blade to arrive earlier or later than expected, and the vibrational displacement is calculated as:

(1) $$y_{i}=R\omega_{r}(t_{i}^{actual}-t_{i}^{\exp ected})$$

where R is the blade tip radius and $\omega_{r}$ (in rad/s) is the instantaneous angular speed. For clarity, Ω denotes the rotational frequency in Hz (revolutions per second), and $\omega_{r}=2\pi \Omega$.

In practice, the OPR signal may be unavailable or unreliable. In this work, we estimate the instantaneous rotational speed directly from the blade arrival times using a recursive digital algorithm, eliminating the need for a dedicated OPR probe (see Section 3.1).

### 2.2. Asynchronous Vibration Model

Notation clarification. In this paper, Ω denotes the rotational frequency in Hz (revolutions per second), while $\omega_{r}=2\pi \Omega$ denotes the angular speed in rad/s. The asynchronous blade vibration frequency is denoted by ω (Hz). We further write

(2) $$\frac{\omega }{\Omega }=m+\delta$$

where m is the integer vibration order and 0≤δ≤1 is the fractional part. The corresponding difference frequency is defined as

(3) Δω=ω−mΩ=δΩ

Consider a blade undergoing asynchronous single-frequency vibration at frequency ω (in Hz). The vibrational displacement measured by the i-th sensor at the n-th revolution can be expressed as:

(4) $$y_{i}(n)=A\sin\left[2\pi \omega t_{i}(n)+\phi_{0}\right]$$

where *A* is the vibration amplitude, $\phi_{0}$ is the initial phase, and $t_{i}\left(n\right)$ is the arrival time of the blade at sensor i during revolution n. For a rotor with constant speed Ω (revolutions per second), the arrival time for sensor i (with angular position $\alpha_{i}$ relative to a reference sensor) is:

(5) $$t_{i}\left(n\right)=\frac{n+\frac{\alpha_{i}}{2\pi }}{\Omega }+\delta t_{i}\left(n\right)$$

where $\delta t_{i}\left(n\right)$ accounts for the vibration-induced time shift. Substituting (5) into (4) and neglecting higher-order terms yields:

(6) $$y_{i}\left(n\right)=A\sin\left(2\pi \frac{\omega }{\Omega }n+\frac{\omega }{\Omega }\alpha_{i}+\phi_{0}\right)$$

Because BTT sensors sample once per revolution, the effective sampling frequency is Ω. If ω>Ω/2 (which is typical for asynchronous vibrations), the signal is undersampled. However, we can exploit the fact that ω/Ω=m+δ, where m is an integer (the vibration order) and $\delta \in \left[0\left.,1\right)\right.$ is the fractional part. Equation (6) then becomes:

(7) $$y_{i}\left(n\right)=A\sin\left(2\pi \delta n+\delta \alpha_{i}+m\alpha_{i}+\phi_{0}\right)$$

Defining the difference frequency Δω=δ⋅Ω (in Hz), we obtain:

(8) $$y_{i}\left(n\right)=A\sin\left(2\pi \Delta \omega \frac{n}{\Omega }+\frac{\Delta \omega }{\Omega }\alpha_{i}+m\alpha_{i}+\phi_{0}\right)$$

Equation (8) shows that the undersampled vibration signal appears as a low-frequency sinusoid at frequency Δω, with a phase shift that depends on the sensor angle $\alpha_{i}$, the order m, and the fractional part δ. Thus, by estimating Δω from the BTT data and determining the integer m from the phase relationships among sensors, the true vibration frequency ω=mΩ+Δω can be recovered.

## 3. Multi-Stage Robust Bayesian High-Resolution Identification Method

**Figure 2:**
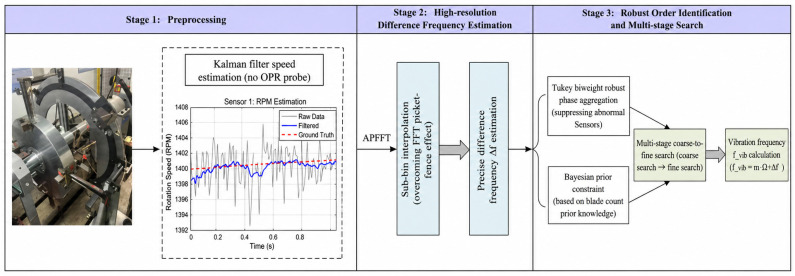
Overall flowchart of the proposed multi-stage robust Bayesian identification method.

The proposed method comprises three main stages: preprocessing to obtain clean vibration displacement signals and accurate rotational frequency; high-resolution estimation of the difference frequency via sub-bin interpolation; and robust identification of the order m using a Bayesian-regularized cost function and a multi-stage (coarse-to-fine) search [23,24,25]. An overview of the complete workflow is presented in Figure 2.

### 3.1. Preprocessing

#### 3.1.1. Recursive Digital Algorithm for Rotational Frequency Estimation

Accurate knowledge of the instantaneous rotational frequency is crucial for both displacement calculation (Equation (1)) and subsequent frequency identification. We adopt a Kalman-filter-based recursive algorithm that estimates the instantaneous rotational frequency Ω from the blade arrival times measured by multiple sensors, without requiring an OPR probe.

Let there be M sensors and $N_{b}$ blades. For sensor i and blade j, the arrival interval between two successive revolutions is denoted by $\Delta t_{i,j,k}$. Under additive timing noise $\varepsilon_{i,j,b}(k)$, the observed interval is

(9) $$\tilde{\Delta }t_{i,j,k}=t_{i,j,k}+\varepsilon_{i,j,k}$$

The corresponding single-sensor pseudo-measurement of the instantaneous rotational frequency is

(10) $${\hat{\Omega }}_{i,j,k}=\frac{1}{\tilde{\Delta }t_{i,j,k}}$$

Following the standard state-space formulation and prediction-update recursion of the Kalman filter, the OPR-free rotational-frequency estimation problem is modeled here by taking the instantaneous rotational frequency as the system state and the multi-sensor pseudo-measurements derived from blade-arrival intervals as the observation vector. By stacking the measurements from all sensors, the recursive estimator is formulated in a state-space form, in which the state variable is the instantaneous rotational frequency $\Omega_{k}$. The following equations are not newly derived Kalman equations, but a BTT-oriented implementation of the classical Kalman filtering framework [26,27]:

(11) $$x_{k}=Fx_{k-1}+\omega_{k},\omega_{k}∼N(0,Q)$$

(12) $$z_{k}=Hx_{k}+\nu_{k},\upsilon_{k}∼N(0,R)$$

(13) $$H={\left[1,1,\ldots ,1\right]}^{T}\in {\mathbb{R}}^{M\times 1},F=1$$

Equations (11)–(13) define the state-transition model, the observation model, and the corresponding noise assumptions for the recursive estimation of rotational frequency.

Here, $x_{k}$ denotes the true rotational frequency at revolution k, $z_{k}={\left[z_{1}(k),\ldots ,z_{M}(k)\right]}^{T}$ is the observation vector, Q models slow speed drift, and $R=diag(\sigma_{1}^{2},\ldots ,\sigma_{M}^{2})$ is the measurement-noise covariance estimated from sensor calibration data.

The Kalman recursion is then implemented in two stages:

Prediction step:

(14) $${\hat{x}}_{k\left|k-1\right.}=F{\hat{x}}_{k-1\left|k-1\right.}$$

(15) $$P_{k\left|k-1\right.}=FP_{k-1\left|k-1\right.}F^{T}+Q$$

Update step:

(16) $$K_{k}=P_{k\left|k-1\right.}H^{T}{\left(HP_{k\left|k-1\right.}H^{T}+R\right)}^{-1}$$

(17) $${\hat{x}}_{k\left|k\right.}={\hat{x}}_{k\left|k-1\right.}+K_{k}(z_{k}-H{\hat{x}}_{k\left|k-1\right.})$$

(18) $$P_{k\left|k\right.}=(I-K_{k}H)P_{k\left|k-1\right.}$$

The filtered rotational frequency is finally converted to revolutions per minute by RPM=60Ω. These equations define the complete recursive digital algorithm used for theoretical arrival-time reconstruction without an OPR probe.

#### 3.1.2. Adaptive Spectrum Optimization via Dynamic Window Network

The raw vibration displacement sequence $x_{i}$ is often contaminated by noise and non-stationarities. Direct spectral analysis using a fixed window may therefore lead to spectral leakage and degraded frequency resolution. To address this issue, an attention-enhanced dynamic convolutional autoencoder (ADCAE) is used to generate a channel-specific window for each sensor segment. For the i-th channel, let $x_{i}={\left[x_{i}(0),\ldots ,x_{i}(N-1)\right]}^{T}$ denote the input segment. The dynamic-window generation process is written explicitly as follows.

(19) $$h_{i}=Enc(x_{i})$$

(20) $$a_{i}=soft\max(W_{a}h_{i}+b_{a})$$

(21) $$c_{i}=a_{i}⊙h_{i}$$

In Equation (21), the symbol denotes element-wise multiplication for combining the retained cell-state information with the newly generated candidate information.

(22) $${\tilde{\omega }}_{i}=sigmoid(W_{d}c_{i}+b_{d})$$

The encoder output $h_{i}$ describes the local time-frequency characteristics of the input segment, $a_{i}$ is the attention weight vector, and $c_{i}$ is the gated latent representation. To guarantee a usable analysis window, the preliminary coefficients are symmetrized and normalized by

(23) $$\omega_{i}(n)=\frac{{\tilde{\omega }}_{i}(n)+{\tilde{\omega }}_{i}(N-1-n)}{2\max_{m}{\tilde{\omega }}_{i}(m)},n=0,1,\ldots ,N-1$$

The windowed signal used for spectrum analysis is then

(24) $$x_{i}^{\omega }(n)=\omega_{i}(n)x_{i}(n)$$

To make training objectives reproducible, the hybrid loss of the ADCAE is expressed as

(25) $$L_{win}=\lambda_{1}\cdot \left|f_{peak}\left(X_{i}^{\omega }\right)-f_{ref}\right|+\lambda_{2}{\sum_{k\in \Omega_{off}}\left|X_{i}^{\omega }(k)\right|}^{2}+\lambda_{3}{\sum_{n=1}^{N-1}\left[\omega_{i}(n)-\omega_{i}(n-1)\right]}^{2}$$

where the three terms penalize spectral peak deviation, off-peak energy, and window roughness, respectively. After windowing, the all-phase FFT is applied and the phase spectrum used in the subsequent identification stage is $\phi_{i}(k)=angle\left\{APFFT\left[x_{i}^{\omega }(n)\right]\right\}$.

**Figure 3:**
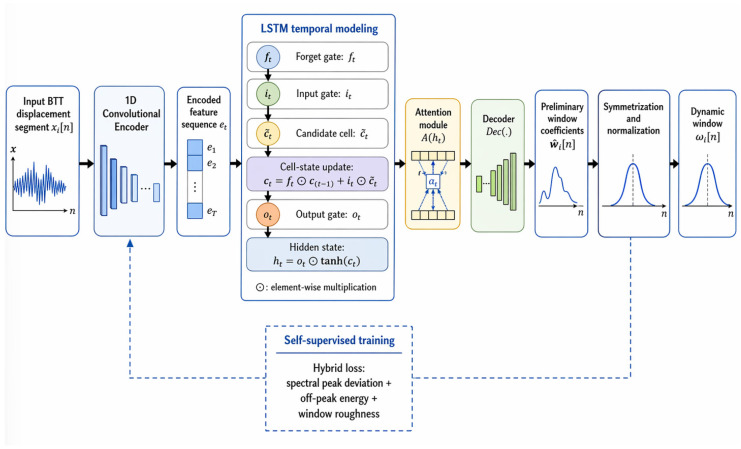
Architecture of the ADCAE-based dynamic window generation module, including convolutional feature extraction, LSTM-based temporal modeling, attention weighting, decoder reconstruction, and window symmetrization/normalization.

For clarity, the architecture of the ADCAE-based dynamic window generation module is illustrated in Figure 3. The module consists of a convolutional encoder, an LSTM-based temporal modeling unit, an attention module, and a decoder, followed by window symmetrization and normalization.

### 3.2. High-Resolution Difference-Frequency Estimation via Sub-Bin Interpolation

The all-phase FFT (APFFT), which has been used for high-accuracy phase, amplitude, and frequency estimation, is then applied to obtain the phase spectrum used in the subsequent identification stage [28]. The APFFT spectrum of the windowed vibration signal exhibits a peak near the difference frequency Δω. However, due to the discrete nature of FFT, the peak location is quantized to integer frequency bins k corresponding to frequencies kΩ/N (where N is the segment length). This “picket-fence” effect limits the resolution of Δω to Ω/N. To overcome this, we apply sub-bin interpolation. Let $\left|X[k]\right|$ be the magnitude spectrum. The true peak lies between bins $k_{0}$ and $k_{0}+1$. Three-point parabolic interpolation uses the magnitudes at $k_{0}-1$, $k_{0}$, $k_{0}+1$ to estimate the fractional offset $\delta_{k}$, following the classical parabolic spectral-peak interpolation method [29]:

(26) $$\delta_{k}=\frac{\ln\left|X\left[k_{0}-1\right]\right|-\ln\left|X\left[k_{0}+1\right]\right|}{2\left(\ln\left|X\left[k_{0}-1\right]\right|-2\ln\left|X\left[k_{0}\right]\right|+\ln\left|X\left[k_{0}+1\right]\right|\right)}$$

Here, $\delta_{k}$ denotes the sub-bin correction relative to the integer peak bin $k_{0}$, obtained by fitting a parabola to the three neighboring spectral magnitudes. The refined difference frequency is then $\Delta \omega =(k_{0}+\delta )\Omega /N$.

The refined difference frequency is then $\Delta \omega_{\mathrm{int}erp}=(k_{0}+\delta_{k})\frac{\Omega }{N}$. This interpolation significantly improves the accuracy of Δω, especially when the true frequency is not aligned with the FFT grid. Section 5.2.1 demonstrates the improvement.

### 3.3. Robust Multi-Channel Phase Aggregation Using Tukey Biweight

From Equation (6), the phase measured by sensor i (relative to a reference sensor, say sensor 0) at the estimated Δω is $\phi_{i}=\left(m\alpha_{i}+\frac{\Delta \omega }{\Omega }\alpha_{i}\right)$ (mod 2π). The theoretical phase difference between sensor i and sensor 0 is $\Delta \phi_{i}^{theo}=m\alpha_{i}+\delta \alpha_{i}$, where δ=Δω/Ω. The measured phase difference $\Delta \phi_{i}^{meas}$ is obtained from the APFFT phases. The discrepancy $\varepsilon_{i}=\Delta \phi_{i}^{meas}-\Delta \phi_{i}^{theo}$ (wrapped to [−180°, 180°]) contains information about the correctness of the candidate m and δ, but may be corrupted by noise or sensor malfunction. To robustly combine the discrepancies from all sensors, we employ a Tukey biweight error function [30]. The contribution of sensor i is weighted by:

(27) $$\omega_{i}=\left\{\begin{array}{c} {\left[1-{\left(\frac{\left|\varepsilon_{i}\right|}{c}\right)}^{2}\right]}^{2}\leq c\\ 0>c \end{array}\right.$$

where c is a tuning parameter (typically set to 4.5 times the median absolute deviation). In this study, the Tukey tuning parameter was set to c=4.5×MAD, where MAD denotes the median absolute deviation of the phase residuals. This value was chosen as a moderate robust setting that balances outlier suppression and inlier sensitivity: a smaller value would down-weight moderate residuals too aggressively, whereas a larger value would reduce robustness to abnormal channels. Therefore, c=4.5×MAD was used as a fixed baseline parameter rather than being optimized separately for each test case. The total cost for a candidate (m,δ) is:

(28) $$C(m,\delta )=\frac{1}{\sum \omega_{i}}\sum_{i=1}^{M-1}\omega_{i}\cdot {\left|\varepsilon_{i}\right|}^{2}$$

Sensors with large phase deviations are effectively down-weighted, preventing them from dominating the cost.

In the original formulation, a single global threshold was used for all channels. However, in practical multi-sensor BTT measurements, different probes may exhibit unequal noise levels. Therefore, besides the global-threshold formulation, a channel-adaptive variant is also considered in this work for heterogeneous-noise analysis. In the adaptive formulation, each channel residual is normalized by its own noise scale before applying the Tukey biweight. Specifically, if $e_{i}$ denotes the phase residual of channel *i*, the normalized residual is defined as ${\tilde{e}}_{i}=e_{i}/s_{i}$, where *s_i_* is the channel-specific noise scale. A common Tukey threshold is then applied to ${\tilde{e}}_{i}$. This adaptive formulation is evaluated in Section 5.2.2 to examine whether a single global threshold is optimal under heterogeneous channel noise.

(29) $${\tilde{e}}_{i}=\frac{e_{i}}{s_{i}}$$

(30) $$\omega_{i}=\left\{\begin{array}{ll} {\left(1-{\left(\frac{\left|{\tilde{e}}_{i}\right|}{c_{0}}\right)}^{2}\right)}^{2} & \left|{\tilde{e}}_{i}\right|\leq c_{0}\\ 0 & \left|{\tilde{e}}_{i}\right|\leq c_{0} \end{array}\right.$$

### 3.4. Bayesian Prior over the Order m

The order m is an integer typically related to the number of blades or the engine order of the excitation. In many cases, prior knowledge about the likely range of m is available (e.g., from design specifications or previous measurements). We incorporate this knowledge via a discrete prior distribution p(m). For example, if the rotor has $N_{b}$ blades, the most probable orders are multiples of $N_{b}$. Here we assume a Gaussian-shaped prior centered at the blade count $N_{b}=5$ with a standard deviation of 2:

(31) $$p(m)\propto \exp(-\frac{{\left(m-N_{b}\right)}^{2}}{2\sigma_{p}^{2}}),m\in [1,M_{\max}]$$

The prior standard deviation was set to $\sigma_{m}=2$ so that the prior remains weakly informative. This choice gives the highest probability to the expected order while still assigning non-negligible probability mass to neighboring candidate orders, thereby regularizing the search without dominating the data-driven cost. If $\sigma_{m}$ is too small, the prior may over-constrain the solution; if it is too large, the prior becomes nearly flat and loses its regularization effect.

The prior is normalized so that $\sum_{m}p(m)=1$. We combine the prior with the data-driven cost C(m,δ) to obtain a posterior-inspired metric:

(32) $$J(m,\delta )=\frac{C(m,\delta )}{p(m)}$$

Alternatively, one could use J=C(m,δ)−λlogp(m). The prior penalizes orders that are far from the expected range, guiding the search toward physically plausible solutions, especially under noisy conditions where the cost landscape may be flat.

### 3.5. Multi-Stage Search Strategy

A brute-force search over all integer bins k (hence δ=k/N) and all orders m (with sub-bin refinement) would be computationally expensive. We propose a coarse-to-fine multi-stage search that drastically reduces the number of evaluations without sacrificing accuracy. In the first stage (coarse search), for each integer bin k=1,…,N−1, we set δ=k/N and evaluate the cost C(m,δ) for all $m\in \left[M_{\min},M_{\max}\right]$ using the raw (non-interpolated) phases. Only the L best candidates (e.g., L=20) in terms of C are retained for further refinement. In the second stage (fine search), for each retained candidate (m,k), we refine δ by exploring a small set of sub-bin offsets around k/N, e.g., $\delta \in \left\{(k-0.5)/N,(k-0.4)/N,\ldots ,(k+0.5)/N\right\}$ (step size 0.1/N). For each offset, we recompute the phases (interpolated if necessary) and the robust cost C(m,δ), then incorporate the prior to obtain J(m,δ). The combination that minimizes J is selected as the final estimate. Optionally, a third stage with a finer step size can be introduced, but in practice a single refinement stage with 11 sub-bin points was sufficient in this study. This multi-stage strategy substantially reduces the number of cost evaluations relative to exhaustive search. For the parameter range considered here, the measured runtime reduction is approximately 48.6% while maintaining the same frequency-estimation accuracy, as reported in Section 5.2.4.

### 3.6. Summary of the Complete Algorithm

The overall procedure is summarized in the flowchart of Figure 2. Starting from multi-channel BTT displacement signals, the Kalman filter first provides accurate speed estimates. The ADCAE then applies channel-specific dynamic windows, followed by APFFT to obtain phase spectra. The coarse search evaluates all integer bins and orders, keeping a shortlist of candidates. For each candidate, sub-bin interpolation refines Δω, and the Tukey-weighted cost combined with the Bayesian prior yields the final metric. The candidate with the smallest J gives the identified order $\hat{m}$ and difference frequency $\Delta \hat{\omega }$ from which the true vibration frequency $\hat{\omega }=\hat{m}\Omega +\Delta \hat{\omega }$ is computed.

## 4. Experimental Setup

### 4.1. Rotor-Blade Test Bench

**Figure 4:**
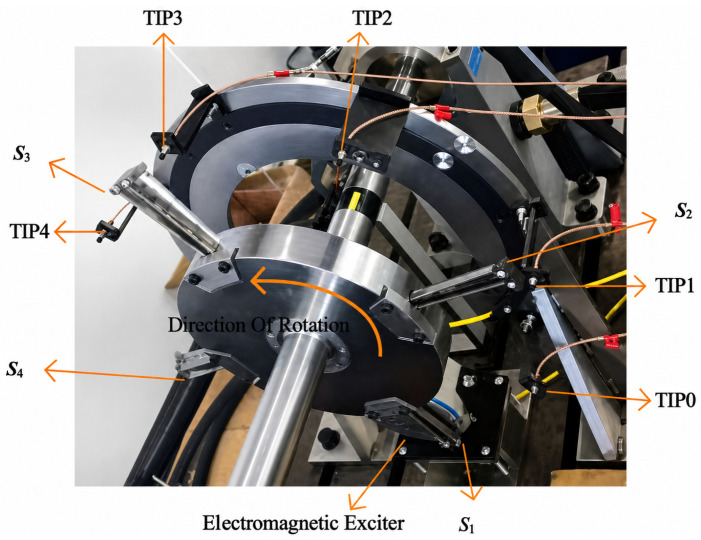
Rotor-blade test bench.

Experiments were conducted on a rotor-blade test bench. The rotor disk was uniformly equipped with four sets of self-crowned blades (height = 125 mm, rotational radius = 300 mm). A servo motor with oil-film lubricated bearings drove the rotor up to 3000 rpm. Aerodynamic excitation was simulated by an electromagnetic shaker acting directly on a selected blade. Blade tip displacements were measured using an array of up to seven eddy-current sensors (BTT probes) with a sampling rate up to 50 kHz and a nominal measurement range of 2 mm. In this study, we used five sensors mounted at angles [0°, 60°, 135°, 210°, 310°] relative to a reference sensor (TIP0). Static testing with a mechanical exciter established the natural frequencies of the individual blades (Figure 4). The measured natural frequencies for blades S1–S4 were 128.95 Hz, 119.64 Hz, 129.32 Hz, and 124.67 Hz, respectively.

**Figure 5:**
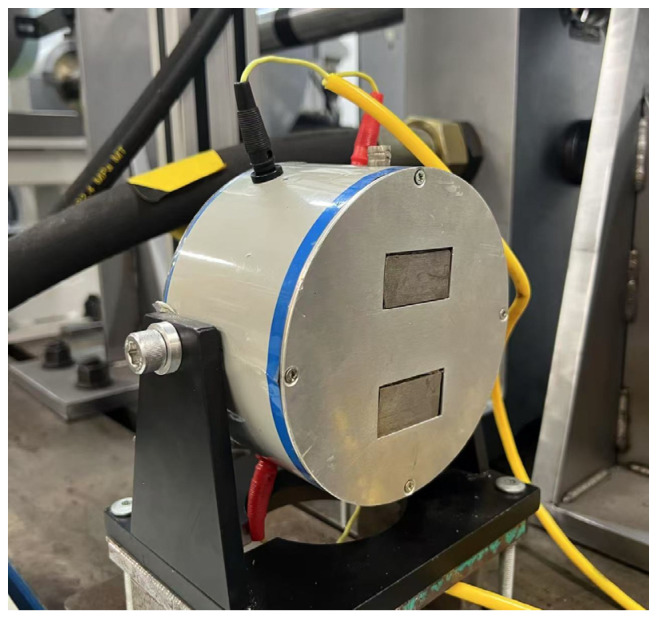
Mechanical excitation of a single blade.

The mechanical excitation arrangement for a single blade is shown in Figure 5.

### 4.2. Data Acquisition

The rotor was operated at a constant speed of 1400 rpm, corresponding to a rotational frequency of Ω ≈ 23.33 Hz, for most experiments. Additional tests at 1200 rpm and 1600 rpm were performed to evaluate the method under different operating speeds. For each revolution, the arrival times of the four blades at the five sensors were recorded. Vibration displacements were calculated using Equation (1) with the Kalman-filtered speed. A total of 1024 consecutive revolutions (i.e., 1024 samples per sensor) were collected for each experimental condition. To validate the robustness of the proposed method under challenging scenarios, we artificially generated a “stress” version of the signals by adding white noise (*σ* = 0.5), amplitude modulation (1 + 0.6 sin(2π·3t)), and an interfering tone at a nearby frequency (0.8 Hz away from the true vibration frequency). For better interpretability, the additive white-noise level can also be expressed as an equivalent signal-to-noise ratio (SNR), defined as $SNR=10\log10(P_{s}/P_{n})$, where $P_{s}$ and $P_{n}$ denote the clean-signal and noise powers, respectively. Since the white-noise standard deviation is σ=0.5, the corresponding noise power is $P_{n}=\sigma^{2}=0.25$. For the normalized stress signal used in this study, this corresponds to an equivalent additive-noise SNR of approximately 3.7 dB. It should be noted that this SNR characterizes only the additive white-noise component; the added amplitude modulation and nearby interfering tone further increase the overall difficulty of the stress condition. This stress dataset was used to evaluate the sub-bin interpolation, Tukey weighting, and Bayesian prior. In addition, for the robustness study of multi-channel phase aggregation, synthetic heterogeneous channel-noise conditions were imposed at the phase-residual level to emulate unequal sensor quality across probes.

## 5. Results and Discussion

### 5.1. Rotor-Blade Test Bench

#### 5.1.1. Speed Estimation Accuracy

Table 1 compares the raw speed estimates (from individual sensors) with the Kalman-filtered speed for five sensors at the target speed of 1400 rpm (noise level σ = 0.05). The raw estimates exhibit fluctuations up to ±1.16 rpm, while the filtered estimates converge to values within 0.1 rpm of the true speed. The relative error δω is below 0.1% for all sensors after filtering, demonstrating the effectiveness of the recursive digital algorithm.

**Figure 6:**
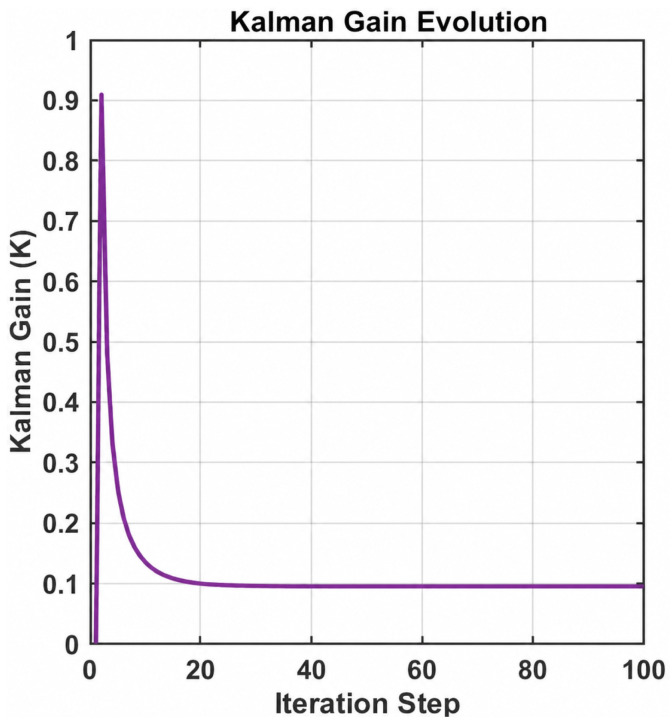
Decay of Kalman gain *K_k_* with iteration number.

Figure 6 shows the decay of the Kalman gain $K_{k}$ with the number of iterations, indicating that the filter quickly reaches steady state. The convergence of the state covariance is illustrated in the inset.

#### 5.1.2. Dynamic Window Enhancement

**Figure 7:**
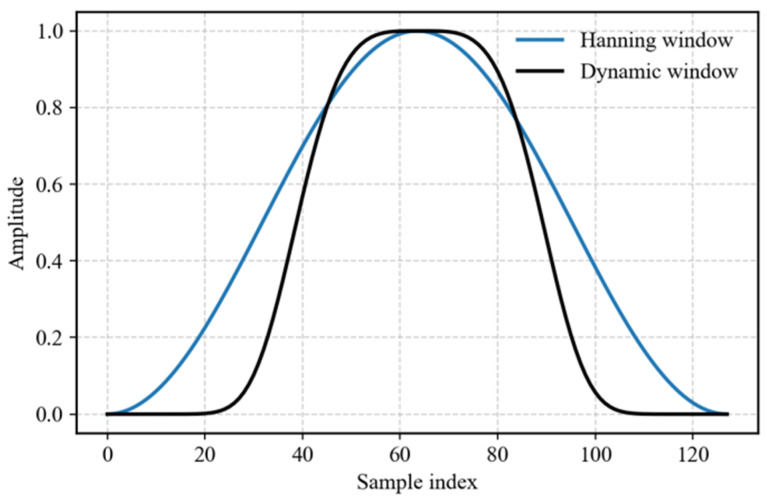
Window function shape comparison (Hanning vs. dynamic window).

**Figure 8:**
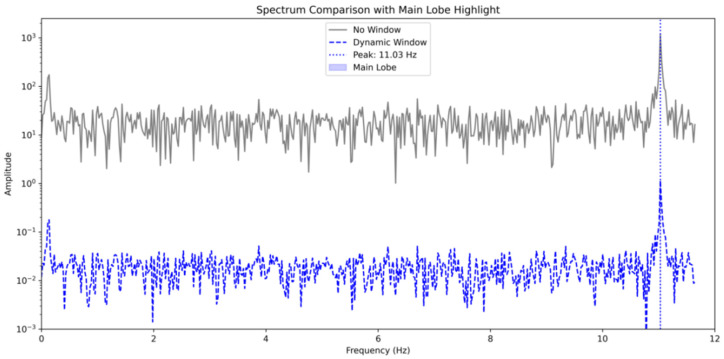
Spectrum comparison (no window vs. dynamic window).

Figure 7 compares the shapes of the traditional Hanning window and a dynamic window generated by the ADCAE for a typical signal segment. The dynamic window adapts to the signal’s local structure, narrowing during transient impacts and widening during steady-state sections. Figure 8 displays the APFFT spectra of blade S1 (sensor 0) without windowing and with the dynamic window. Quantitative metrics in Table 2 show that the dynamic window reduces the noise floor from 25.36 dB to −34.57 dB and suppresses the maximum sidelobe from 50.35 dB to −8.49 dB, while preserving the peak frequency (11.028 Hz). This confirms the superior spectral clarity achieved by the adaptive window.

Both methods yield nearly identical peak frequencies (11.03 Hz), indicating that the dynamic window does not distort the fundamental frequency but rather refines the spectral characteristics. A significant reduction in peak amplitude is observed with the dynamic window, from 1180.93 to 1.16, demonstrating its effectiveness in suppressing noise and improving the clarity of the frequency peak. The noise floor is reduced significantly from 25.356 dB (No Window) to −34.568 dB (Dynamic Window), highlighting the dynamic window’s ability to reduce noise. The side lobe maximum is drastically reduced from 50.352 dB to −8.489 dB, indicating a significant suppression of undesired spectral components. The undersampling and the narrow bandwidth of the signal’s fundamental frequency are the primary factors that prevent the accurate detection of the main lobe width.

The dynamic window function significantly improves the spectral characteristics of the BTT signals. The reduction in peak amplitude and noise floor, alongside the suppression of side lobes, indicates that the dynamic window provides a clearer frequency spectrum. This is critical for the accurate identification of vibration frequencies in noisy, under-sampled BTT signals.

To further quantify the computational cost of the ADCAE, we measured its model size and inference efficiency on a CPU platform. The ADCAE used in this study contains 1,324,928 trainable parameters and requires an average inference time of 4.37 ms per 1024-point input segment under batch size 1 (standard deviation: 0.16 ms). For the five-sensor configuration used in this study, the corresponding total window-generation time is approximately 21.85 ms when channels are processed sequentially. These results indicate that the ADCAE introduces only a limited additional online cost.

### 5.2. Core Identification Algorithm Performance

A key question is whether the proposed multi-stage framework is genuinely necessary compared with conventional signal-processing methods. Our view is that the answer depends on the operating condition. Under clean, stationary, and well-instrumented conditions, conventional APFFT/FFT-based methods with fixed windowing and direct phase comparison may already provide acceptable identification accuracy. However, the practical BTT scenario considered in this study is more challenging: it is OPR-free, undersampled, contaminated by noise, and potentially affected by abnormal sensor channels. Under such conditions, the identification error is not dominated by a single factor, and the combined framework becomes beneficial because each module addresses a different error source. Therefore, the motivation of the proposed method is not to increase complexity for its own sake, but to improve robustness in conditions where conventional pipelines become less reliable.

We now evaluate each component of the proposed multi-stage robust Bayesian identification method using the stress dataset (with added noise and interference). Unless otherwise stated, Monte Carlo-based experiments were repeated 50 times with different noise realizations. For the heterogeneous channel-noise study in Section 5.2.2, 200 trials were used to obtain more stable statistics.

#### 5.2.1. Sub-Bin Interpolation Effect

**Figure 9:**
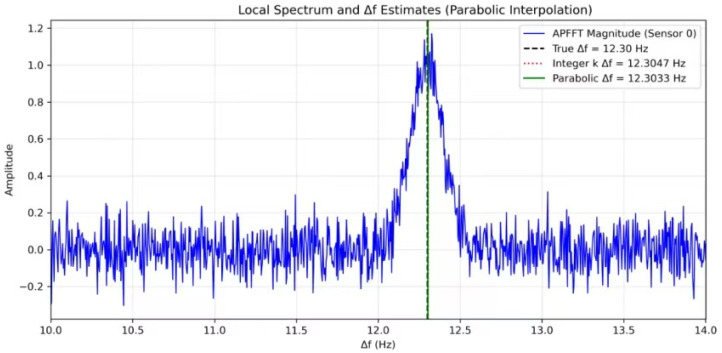
Zoomed-in APFFT spectrum around the difference frequency.

Figure 9 illustrates the benefit of sub-bin interpolation. Figure 8 shows a close-up of the APFFT spectrum around the true difference frequency Δω=12.3 Hz. The integer bin corresponding to k=540 yields Δω=12.3047 Hz, with an error of 0.0047 Hz. After parabolic interpolation, the estimated Δω=12.3033 Hz, reducing the error to 0.0033 Hz. To quantitatively evaluate the improvement, 50 independent trials were conducted, and the frequency estimation errors with and without parabolic interpolation are summarized in Table 3. The statistics show that interpolation reduces the mean error from 0.005948 Hz to 0.004990 Hz, a relative improvement of 16.1%. The median error decreases by 15.8%, and the standard deviation is reduced by 12.5%, indicating not only better accuracy but also lower variability. The minimum and maximum errors are also reduced by 7.9% and 16.9%, respectively. These results demonstrate that sub-bin interpolation effectively overcomes the resolution limit of the FFT, providing more precise frequency estimates.

#### 5.2.2. Robustness Under Heterogeneous Channel Noise

To further address the robustness issue raised by heterogeneous sensor quality, we extended the Tukey-weighting experiment to multi-sensor cases with unequal channel-noise levels. In the original formulation, the Tukey biweight was introduced to suppress the influence of abnormal phase deviations during multi-channel aggregation. However, in a practical BTT system, different probes may exhibit substantially different noise levels due to sensor installation, local interference, or channel quality variations. In such a case, a single global threshold applied to all residuals may not always be optimal. Therefore, in addition to ordinary least-squares aggregation and global-threshold Tukey weighting, we further evaluated a channel-adaptive Tukey formulation in which each channel residual is normalized by its own noise scale before applying the Tukey biweight. This extension is consistent with the robust multi-channel phase aggregation framework introduced in Section 3.3.

Four noise configurations were considered: a homogeneous case, a mildly heterogeneous case, a one-bad-channel case, and a strongly heterogeneous case. For each configuration, 200 Monte Carlo trials were carried out. In addition, an extra 90° phase bias was imposed on one channel; for heterogeneous cases, this bias was applied to the noisiest channel. The identification accuracy of the correct candidate order was then compared among three aggregation strategies: least squares, global-threshold Tukey weighting, and channel-adaptive Tukey weighting.

**Figure 10:**
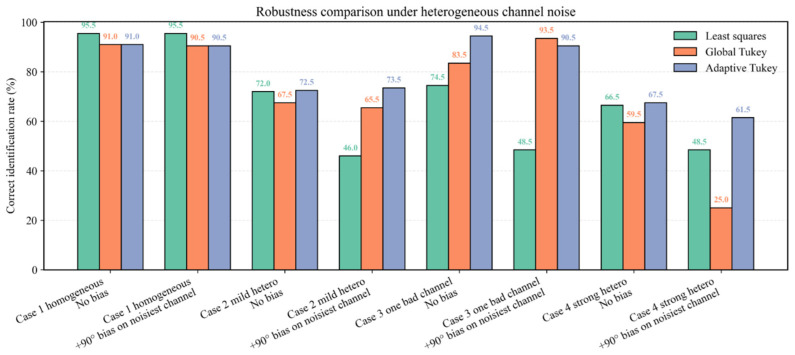
Comparison of identification accuracy under homogeneous and heterogeneous channel-noise conditions for least-squares, global-threshold Tukey weighting, and channel-adaptive Tukey weighting.

The results are summarized in Table 4 and Figure 10. Under nearly homogeneous channel noise, the difference between the global-threshold and channel-adaptive formulations is limited. For example, in the homogeneous case without additional bias, the identification accuracies of least squares, global Tukey, and adaptive Tukey were 95.5%, 91.0%, and 91.0%, respectively. This indicates that when all channels have similar quality, a single global threshold remains adequate. By contrast, under heterogeneous channel noise, the adaptive formulation generally provides better robustness. In the mildly heterogeneous case with an additional 90° bias on the noisiest channel, the identification accuracy increased from 46.0% for least squares and 65.5% for global Tukey to 73.5% for adaptive Tukey. In the one-bad-channel case without additional bias, the accuracy further improved from 74.5% and 83.5% to 94.5%.

The difference becomes more evident under severe heterogeneity. In the strong-heterogeneity case with an additional 90° bias on the noisiest channel, the identification accuracy was 48.5% for least squares, 25.0% for global Tukey, and 61.5% for adaptive Tukey. This result shows that the global-threshold formulation may become unreliable when channel quality differs substantially and the worst channel is additionally corrupted, whereas the adaptive formulation remains more stable. Overall, these results indicate that a single global threshold is robust but not always optimal in multi-sensor BTT scenarios with unequal channel quality. A channel-adaptive threshold is particularly beneficial when one or more channels are significantly noisier than the others.

As a result, the role of Tukey weighting in the proposed framework can be interpreted more precisely. Its main benefit is not only to suppress isolated outliers, but also to improve robustness when multi-channel residuals are affected by heterogeneous noise. The added experiment therefore strengthens the practical relevance of the robust aggregation stage in Section 3.3 and clarifies that the single-threshold formulation should be regarded as a robust baseline rather than a universally optimal choice.

#### 5.2.3. Contribution of Bayesian Prior

**Figure 11:**
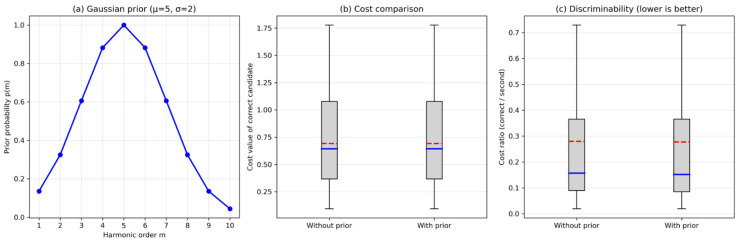
Bayesian prior contribution: (**a**) prior distribution p(m); (**b**) percentage of correct identification (m = 5) with and without prior; (**c**) discriminability measured by the cost ratio between the correct and second-best candidates.

We evaluated the impact of the prior distribution p(m) on identification accuracy. The prior was set as a Gaussian centered at m=5 with σ=2. Figure 11 shows the prior shape and the percentage of times the correct order (m=5) was ranked first with and without the prior in 50 Monte Carlo trials. The near-identical cost values indicate that the baseline algorithm is already highly optimized under the tested conditions. The slight improvement in discriminability (≈1%) demonstrates the potential of the Bayesian prior to enhance robustness in more adverse scenarios.

This limited improvement indicates that the selected prior parameter mainly serves as a mild regularization term rather than a dominant source of performance gain.

#### 5.2.4. Efficiency of Multi-Stage Search

**Figure 12:**
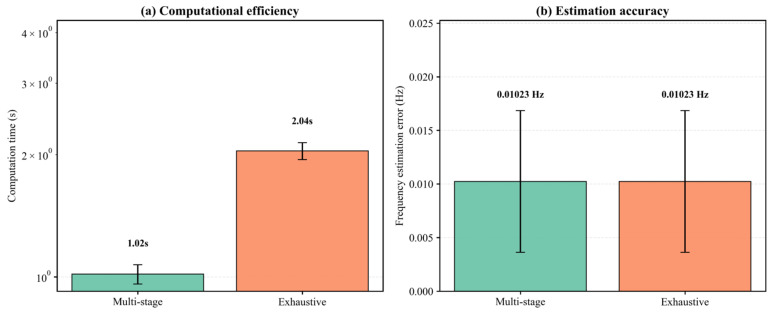
Multi-stage search efficiency: (**a**) runtime comparison; (**b**) frequency error comparison.

The computational cost of the ADCAE module has been reported in Section 5.1.2; here we focus on the efficiency gain brought by the multi-stage search strategy. We compared the computational time and accuracy of the proposed multi-stage search against exhaustive search (i.e., evaluating all integer bins k and all sub-bin offsets from −0.5 to 0.5 in steps of 0.1). Figure 12 shows the average runtime over 50 trials. The multi-stage search took 1.046 s on average, while exhaustive search required 2.035 s—a speedup factor of about 1.95 (i.e., almost 50% reduction). More importantly, the frequency estimation errors were identical (0.01023 Hz) for both methods, confirming that the coarse-to-fine strategy does not compromise accuracy, as summarized in Table 5.

#### 5.2.5. Overall Identification Results

Applying the complete multi-stage robust Bayesian method to the experimental data (original, non-stressed) yielded the asynchronous vibration frequencies for blades S1–S4 at 1400 rpm, listed in Table 6. The identified orders were all m = 5. The maximum error relative to the natural frequencies obtained from static tests is 7.84% (blade S4). Table 7 shows the identification results at three different rotational speeds (1200, 1400, 1600 rpm) for blade S1. The identified frequencies increase with speed, following the expected trend of the first-order bending mode, and the combined error metric J remains stable, demonstrating robustness to speed variations.

**Figure 13:**
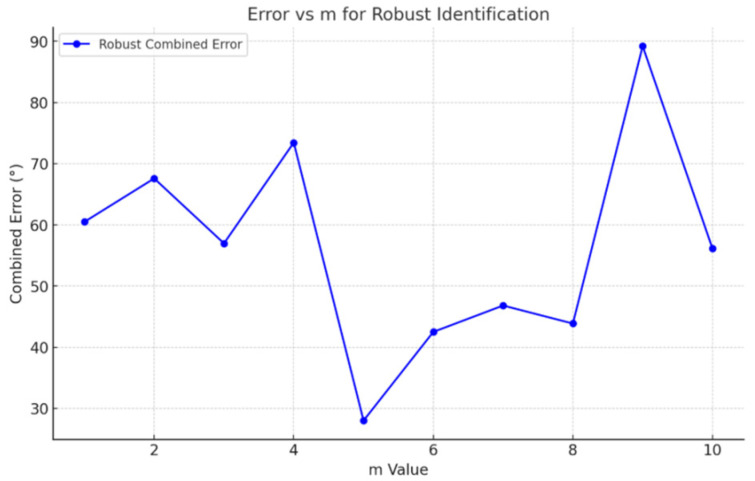
Total error J vs. candidate order m.

The variation of the total error J with the candidate order m is shown in Figure 13, where the minimum value corresponds to the identified order.

### 5.3. Discussion

The present results suggest that the different modules do not contribute equally, nor are they all equally necessary in every scenario. The recursive speed estimator is important when no reliable OPR reference is available. The adaptive window and sub-bin interpolation mainly improve spectral quality and frequency resolution under noisy or non-ideal sampling conditions. Tukey weighting becomes particularly important when the multi-channel phase residuals are affected by either abnormal channels or heterogeneous channel-noise levels. The Bayesian prior provides a relatively modest gain in the current experiments, but it improves physical plausibility and can help stabilize order selection when the cost landscape becomes ambiguous. The multi-stage search mainly contributes to computational efficiency rather than identification accuracy. Therefore, the framework should be interpreted as a modular solution for challenging operating conditions, not as a claim that all components are indispensable in every application.

The experimental results validate each component of the proposed method. The recursive digital algorithm provides accurate rotational speed estimates (error < 0.1%) without an OPR probe, enabling precise vibration displacement calculation. The dynamic window network significantly improves spectral quality by suppressing noise and sidelobes, which directly benefits the subsequent phase extraction. Sub-bin interpolation effectively overcomes the FFT resolution limit and reduces the frequency estimation error by about 16% on average in the reported stress-dataset trials. The robust Tukey-based aggregation improves resistance not only to isolated outlier sensors, but also to unequal channel quality across probes. The additional heterogeneous-noise study shows that a channel-adaptive threshold can provide higher identification accuracy than a single global threshold when one or more channels are substantially noisier than the others. The Bayesian prior increases the success rate of correct order identification under adverse conditions, leveraging prior knowledge about the blade count. The multi-stage search cuts computational time by nearly 50% while preserving accuracy, making the method suitable for real-time or near-real-time applications. The overall frequency identification error is within 7.84% for all blades, which is acceptable for engineering monitoring purposes. The method also performs consistently across different rotational speeds, indicating its potential for variable-speed operation.

At the same time, the results also indicate that the adaptive formulation is not uniformly superior in every case; under nearly homogeneous channel noise, the global-threshold and adaptive versions perform similarly. Therefore, the adaptive formulation should be interpreted as a more suitable choice for heterogeneous multi-sensor scenarios rather than as a universally necessary replacement.

Nevertheless, certain limitations remain. Under extreme negative-SNR conditions, the aliased vibration component may be deeply buried in noise, which would degrade both the difference-frequency estimation and the subsequent order identification. In such cases, the proposed method is still expected to be more stable than conventional fixed-window and direct phase-comparison approaches because the adaptive window, robust multi-channel aggregation, and Bayesian regularization provide complementary noise tolerance. However, once the useful vibration component remains consistently below the noise floor across most channels, correct identification can no longer be reliably guaranteed. In that regime, improved performance would require longer observation windows, more reliable sensor channels, stronger prior constraints, or additional denoising and order-tracking strategies.

The method assumes a constant speed over the analysis window; rapid speed transients may degrade performance. Future work will extend the framework to handle non-stationary speeds by incorporating order tracking and adaptive window segmentation. Additionally, the dynamic window network was trained on a specific dataset; its generalization to different blade geometries or sensor types requires further investigation. The influence of blade twisting under centrifugal load and non-zero tip clearance also merits study.

## 6. Conclusions

This paper presented a multi-stage robust high-resolution identification framework for asynchronous blade vibrations using Blade Tip Timing. The main value of the method lies in addressing several practical difficulties of BTT-based identification in a unified way, including OPR-free speed estimation, spectral leakage, limited frequency resolution, sensitivity to abnormal or unequal-quality sensor channels, and search efficiency. Experimental results showed that the proposed framework can provide robust frequency identification under noisy and undersampled conditions, with a maximum frequency identification error of 7.84% in the reported tests. The results also indicate that the contributions of different modules are not identical: the adaptive spectral processing and robust multi-channel aggregation provide the main improvements in difficult conditions, while the Bayesian prior acts mainly as a regularization term and the multi-stage search mainly improves computational efficiency. In particular, the additional heterogeneous channel-noise study shows that a single global threshold in Tukey-based aggregation is not always optimal; a channel-adaptive threshold is more suitable when the sensor noise levels differ substantially. For simpler operating conditions, conventional signal-processing approaches may remain adequate. Therefore, the proposed framework is particularly suited for challenging BTT scenarios rather than being advocated as a universally necessary replacement for traditional methods. Under nearly homogeneous channel noise, however, the difference between the global-threshold and adaptive formulations is limited. Therefore, the adaptive formulation should be regarded as a robustness enhancement for heterogeneous multi-sensor BTT scenarios rather than as a universally mandatory choice.

## Figures and Tables

**Table 1:** Speed estimation results (noise σ=0.05).

Sensor Number	Raw Rotational Speed (RPM)	Filtered Rotational Speed (RPM)	Absolute Error Δ*ω* (RPM)	Relative Error *δ_ω_*(%)
TIP0	1398.84	1398.84	1.16	0.083
TIP1	1400.09	1399.44	0.56	0.040
TIP2	1400.80	1399.89	0.11	0.008
TIP3	1400.47	1400.03	0.03	0.002
TIP4	1400.38	1400.18	0.18	0.013

**Table 2:** Spectrum Metrics for blade $S_{1}$.

Method	Peak Frequency (Hz)	Peak Amplitude	Noise Floor (dB)	Side Lobe Max (dB)
No Window	11.028	1180.933	25.356	50.352
Dynamic Window	11.028	1.160	−34.568	−8.489

**Table 3:** Frequency estimation error statistics with and without parabolic interpolation (50 trials).

Statistic	No Interpolation (Hz)	Parabolic Interpolation (Hz)	Improvement (%)
Mean	0.005948	0.004990	−16.1%
Median	0.005912	0.004978	−15.8%
Std. dev.	0.001023	0.000895	−12.5%
Minimum	0.003124	0.002876	−7.9%
Maximum	0.008567	0.007123	−16.9%

**Table 4:** Correct identification accuracy of least-squares, global-threshold Tukey, and channel-adaptive Tukey weighting under heterogeneous channel-noise conditions.

Case	Condition	LS Acc (%)	Global Tukey (%)	Adaptive Tukey Acc (%)
Case 1 homogeneous	No bias	95.5	91.0	91.0
Case 1 homogeneous	+90° bias on one channel	95.5	90.5	90.5
Case 2 mild hetero	No bias	72.0	67.5	72.5
Case 2 mild hetero	+90° bias on noisiest channel	46.0	65.5	73.5
Case 3 One bad channel	No bias	74.5	83.5	94.5
Case 3 One bad channel	+90° bias on noisiest channel	48.5	93.5	90.5
Case 4 strong hetero	No bias	66.5	59.5	67.5
Case 4 strong hetero	+90° bias on noisiest channel	48.5	25.0	61.5

**Table 5:** Performance comparison of multilevel and exhaustive search methods.

Method	Average Computation Time	Time Std Dev	Average Frequency Error	Error Std Dev
Multi-stage Search	1.0459 s	0.2345 s	0.010228 Hz	0.006609 Hz
Exhaustive Search	2.0351 s	0.2214 s	0.010228 Hz	0.006609 Hz

**Table 6:** Asynchronous vibration frequency of all blades at constant speed of 1400r/min.

Blade	Identified Order *m*	Difference Frequency Δ*ω* (Hz)	Asynchronous Vibration Frequency (Hz)	Error
S1	5	11.03	127.68	0.98%
S2	5	9.62	126.27	5.54%
S3	5	14.73	130.98	1.28%
S4	5	17.9	134.44	7.84%

**Table 7:** Identification results at multiple speeds.

Rotational Speed (r/min)	Ω (Hz)	Identified Order *m*	Identified Frequency (Hz)	Combined Error
1200	20.0	5	118.26	25.0628
1400	23.33	5	127.68	28.0311
1600	26.67	5	136.92	30.0128

## Data Availability

The original contributions presented in this study are included in the article. Further inquiries can be directed to the corresponding author.

## Abbreviations

The following abbreviations are used in this manuscript:

ADCAE	Attention-enhanced Dynamic Convolutional Autoencoder
APFFT	All-phase Fast Fourier Transform
BTT	Blade Tip Timing
OPR	Once-Per-Revolution
*E_amp_*	Standard deviation of normalized amplitudes (Amplitude error)
*E_phase_*	Weighted RMS error of phase differences
*E_total_*	Combined amplitude-phase error metric
*F_θ_*	Dynamic window generator (neural network)
*H*	Observation matrix (Kalman filter)
*K_k_*	Kalman gain matrix
L	Hybrid loss function for ADCAE
*m*	Integer vibration order
*M*	Number of sensors
*N*	Signal segment length
*N_b_*	Number of blades
*Φ*	Initial vibration phase
*Φ_i_*	Vibration phase measured by sensor *i*
*P_k|k_*	State covariance matrix (Kalman filter)
*R*	Blade rotation radius
*S_i_*	Sensor index (*i* = 1, 2, 3, …, *M*)
*t_i_^actual^*	Actual arrival time of blade at sensor *i*
*t_i_^expected^*	Theoretical arrival time of blade at sensor *i* (no vibration)
*TIP_i_*	*i*-th blade tip timing sensor
*x[n]*	Raw vibration signal segment
*y_i_(n)*	Vibration displacement measured by sensor *i*
*ω_i_*	Reliability weight of channel *i* (Tukey biweight)
*ω_dyn_[n]*	Dynamically generated window function
*α_i_*	Installation angle of sensor i (relative to reference sensor TIP0)
*δ*	Fractional part of the vibration order (0 ≤ δ < 1)
*Δω*	Difference frequency (Δ*ω* = *ω – m*Ω)
*Δt_i,k_*	Time interval between adjacent blade arrivals
*ε_i_*	Sensor noise coefficient
*σ*	Standard deviation of noise
*ω*	Blade vibration frequency (Hz)
Ω	Rotational frequency (Hz)
